# Erstes kausal wirkendes Medikament gegen Alzheimer? Die FDA lässt Aducanumab zu

**DOI:** 10.1007/s00115-021-01182-1

**Published:** 2021-09-07

**Authors:** Klaus Hager

**Affiliations:** Institut für Allgemeinmedizin und Palliativmedizin, Medizinische Hochschule Hannover (MHH), Carl-Neuberg-Str. 1, 30625 Hannover, Deutschland

In Deutschland sind die Cholinesterasehemmer sowie Memantin zur Behandlung der Alzheimer-Krankheit zugelassen. Seit fast 20 Jahren hat es keine Neuzulassungen von Medikamenten gegen diese Erkrankung mehr gegeben, was für Patienten wie Ärzte sehr enttäuschend war. Die bisher für die Alzheimer-Krankheit zugelassenen Medikamente besitzen lediglich eine symptomatische Wirkung. Notwendig wäre eine kausale Therapie („disease modifying“), die den Verlauf der Erkrankung tatsächlich aufhalten soll.

## Monoklonale Antikörper

Das derzeit am weitesten entwickelte Therapieprinzip im Rahmen der Amyloid-Hypothese sind monoklonale Antikörper gegen Amyloid-Beta (Abeta [Aß]) im Sinne einer passiven Immunisierung. Amyloid-Beta entsteht im Rahmen des Abbaus des Amyloid-Precursor-Proteins (APP) im Gehirn als 1‑42- oder 1‑40-Beta-Amyloid. Amyloid-Beta hat die Tendenz zu aggregieren, Monomere, Oligomere, Fibrillen und schließlich den Amyloidkern der Alzheimer-Plaques zu bilden. Die Antikörper sollen einerseits dem Immunsystem helfen, das Aß abzubauen, andererseits können sie die Aggregationstendenz von Amyloid beeinflussen, z. B. die Bildung von Oligomeren, denen die größte Neurotoxizität zugeschrieben wird.

Eine Reihe von Antikörper ist schon in Phase-III-Studien geprüft worden. Sie vermindern teilweise den Amyloidgehalt des Gehirns, konnten hingegen die kognitiven Funktionen nur gering verbessern [1]. Für eine Zulassung reichten die Ergebnisse bislang nicht aus [2]. Der letzte Antikörper, der keine ausreichende Wirksamkeit nachweisen konnte, war Solanezumab [3]. Das Deutsche Ärzteblatt berichtete 2016 über die Einstellung der Weiterentwicklung [4].

Die Antikörper unterscheiden sich in verschiedenen Eigenschaften, sodass die Unwirksamkeit eines Antikörpers nicht zwangsläufig bedeutet, dass dies bei einem anderen Antikörper ebenfalls der Fall sein muss.

Typische Nebenwirkungen der Antikörpertherapie sind die sog. ARIAs („amyloid-related imaging abnormalities“), also Effekte, die im Magnetresonanztomogramm (MRT) gesehen werden können. Es werden ARIA‑E und ARIA‑H unterschieden. Bei ARIA‑E tritt ein fokales Hirnödem auf, bei ARIA‑H Mikroblutungen im Gehirn. Die Häufigkeit der ARIAs hängt unter anderem vom jeweiligen Antikörper, dem Durchtritt durch die Blut-Hirn-Schranke und von der Menge des Antikörpers ab.

## Aducanumab

Aducanumab (Aduhelm) ist ein monoklonaler Antikörper der Firmen Eisai und Biogen. In einer Phase-I-Studie wurde eine dosisabhängige Verminderung von Beta-Amyloid (Aβ) in der Positronenemissionstomographie (PET) gefunden [5]. Aufgrund dieser positiven Signale wurde von der U.S. Food and Drug Administration (FDA) ein beschleunigtes Zulassungsverfahren („fast track“) beschlossen und es konnte auf eine Phase-II-Studie verzichtet werden. In 2015 wurden dann zwei identische Phase-III-Studien, die EMERGE- sowie die ENGAGE-Studie, gestartet. Als primärer Endpunkt verwendeten beide Studien eine Verbesserung in der Clinical Dementia Rating Scale-sum of the boxes (CDR-SB).

Im März 2019 führte Biogen eine im Rahmen des Studiendesigns geplante Futility-Analyse durch und stoppte in beiden Studien die Rekrutierung, da der CDR-SB-Score in der Verum- sowie in der Placebogruppe weiter gefallen war (zit. n. [6]). Die Therapie sowie die Verblindung wurden aber weitergeführt und die Daten in der Folge erneut analysiert. Die ENGAGE-Studie zeigte zu diesem Zeitpunkt keinen Vorteil, während die EMERGE-Studie mit der höheren Dosis von 10 mg/kg Körpergeweicht eine signifikante Verbesserung des CDR-SB erbrachte. In beiden Studien konnte in den Amyloid-PET-Analysen eine deutliche Verminderung der zerebralen Amyloidmenge nachgewiesen werden, d. h. das Ziel der Behandlung, die Reduktion des Amyloids im Gehirn, schien also erreicht worden zu sein. In einer Pressemitteilung im Oktober 2019 teilten die Firmen dann mit, dass sie in der höheren Dosierung der EMERGE-Studie doch eine Wirksamkeit sahen, was im Juli 2020 zu einem Zulassungsantrag bei der US Food and Drug Administration (FDA) und bei der Europäischen Arzneimittel-Agentur (EMA) führte.

Bei einem Treffen am 06.11.2020 präsentierten einerseits Reviewer von FDA und Biogen die bisherigen Erkenntnisse aus einem klinischen Blickwinkel sowie andererseits eine Gruppe unabhängiger Reviewer aus einer wissenschaftlich-statistisch geprägten Sicht. Die Ergebnisse von EMERGE und ENGAGE sind bislang zwar noch nicht „peer reviewed“ veröffentlicht, die Transparenz der FDA geht aber soweit, dass die Präsentation von Biogen vor der FDA öffentlich zur Verfügung steht [7]. Hinsichtlich der Wirksamkeit hatte sich nach 78 Wochen unter Aducanumab der CDR-SB als primärer Endpunkt um 22 % weniger verschlechtert als unter Placebo. Die sekundären Endpunkte hinsichtlich der Kognition, der Mini-Mental-Status-Test (MMSE) und die kognitive Subskala der Alzheimer’s Disease Assessment Scale (ADAScog), hatten gegenüber Placebo um 18 % bzw. um 27 % weniger abgenommen als unter Placebo. Bei der Funktionalität ergab sich mit der verwendeten Skala (ADCS-ADL-MCI) eine um 40 % geringere Verschlechterung als unter Placebo und beim Neuropsychiatrischen Inventar in der 10-Item-Version (NPI-10) führte die Behandlung mit dem Verumpräparat zu einer 87 % geringeren Verschlechterung als mit Placebo.

Aus der Darstellung von Biogen [7] geht auch die Häufigkeit der ARIAs hervor. Danach traten in beiden Studien zusammengenommen bei 35,0 % der Probanden ARIA‑E und in 19,1 % ARIA‑H auf [7]. Obwohl viele dieser in der MRT zu detektierenden Nebenwirkungen ohne klinische Symptome blieben, ergibt sich daraus doch, dass eine Reihe von MRT-Kontrollen im Rahmen der Therapie stattfinden werden müssen, was die Kosten der Behandlung erhöhen würde.

In Stellungnahmen hatten sich einige Autoren aus prinzipiellen Erwägungen hinsichtlich des Studiendesigns gegen die Zulassung des Medikamentes geäußert (z. B. [8, 9]), andere sprachen sich u. a. mit Hinweis auf die positiven Ergebnisse in der EMERGE-Studie, die Amyloidreduktion im Gehirn und die Notwendigkeit einer Therapieoption für die Erkrankung für eine Zulassung aus (z. B. [10, 11]).

## Entscheidung der FDA

Der Zulassungsantrag von Biogen bei der FDA wurde nun am 07.06.2021 positiv entschieden [12]. Damit existieren mit Aducanumab ein erstes Medikament mit kausaler Wirkung gegen die Alzheimer-Krankheit und ein Hoffnungsträger, der die Amyloid-Hypothese unterstützt sowie der weiteren Entwicklung von Medikamenten einen starken Schub geben sollte.

## Fazit

Trotz aller Euphorie über diese Entwicklung bleibt in Deutschland die Entscheidung der EMA abzuwarten. Dann müssten Kosten und Nutzen bewertet werden. In der Praxis könnte es sein, dass die Kosten sowie die Nebenwirkungen der Therapie mit Aducanumab bewirken, dass das neue Therapieprinzip erst einmal an wenigen Zentren und nur bei einem beschränkten Patientenklientel eingesetzt werden könnte.

Den vielen Patienten, die nun durch die Presse informiert mit großen Hoffnungen in den Praxen anfragen, wird man sagen müssen, dass die neue Behandlung frühestens nach Zulassung durch die EMA im Verlauf des Jahres und, da die in die EMERGE-Studie eingeschlossenen Patienten ein leichtes Stadium der Erkrankung aufgewiesen hatten, dann auch nur für einen kleinen Teil der Betroffenen in einem frühen Stadium verfügbar sein wird. Das Resümee ist also: Hoffnung ja, aber doch noch etwas Geduld.
